# A highly active mineral-based ice nucleating agent supports *in situ* cell cryopreservation in a high throughput format

**DOI:** 10.1098/rsif.2022.0682

**Published:** 2023-02-08

**Authors:** Martin I. Daily, Thomas F. Whale, Peter Kilbride, Stephen Lamb, G. John Morris, Helen M. Picton, Benjamin J. Murray

**Affiliations:** ^1^ Institute of Climate and Atmospheric Science, School of Earth and Environment, University of Leeds, Leeds LS2 9JT, UK; ^2^ Department of Chemistry, University of Warwick, Gibbet Hill, Coventry CV4 7AL, UK; ^3^ Cytiva, Sovereign House, Cambridge CB24 9BZ, UK; ^4^ Discovery and Translational Science Department, Leeds Institute of Cardiovascular and Metabolic Medicine, School of Medicine, University of Leeds, Leeds LS2 9JT, UK

**Keywords:** cell cryopreservation, ice nucleation, supercooling, heterogeneous ice nucleation, toxicology screening, multiwell plates

## Abstract

Cryopreservation of biological matter in microlitre scale volumes of liquid would be useful for a range of applications. At present, it is challenging because small volumes of water tend to supercool, and deep supercooling is known to lead to poor post-thaw cell viability. Here, we show that a mineral ice nucleator can almost eliminate supercooling in 100 µl liquid volumes during cryopreservation. This strategy of eliminating supercooling greatly enhances cell viability relative to cryopreservation protocols with uncontrolled ice nucleation. Using infrared thermography, we demonstrate a direct relationship between the extent of supercooling and post-thaw cell viability. Using a mineral nucleator delivery system, we open the door to the routine cryopreservation of mammalian cells in multiwell plates for applications such as high throughput toxicology testing of pharmaceutical products and regenerative medicine.

## 1. Introduction

Cryopreservation is the process of cooling biological materials, such as cells and tissues, to cryogenic temperatures to allow long-term storage prior to being thawed and used in a multitude of potential applications [1,2]. There are several cryopreservation methodologies all focused on placing cells in a dormant state at low temperatures from which they can be revived to a state where their viability and functionality are preserved, each with their own technical challenges. A key goal in cryopreservation is the prevention of intracellular ice formation (IIF) that is damaging or even lethal to cells. One group of cryopreservation strategies involves cooling very rapidly with the aim of vitrifying the entire sample [3] (i.e. cryopreservation in the absence of ice), but an alternative technique with great potential is to cool relatively slowly at a controlled rate. In controlled rate freezing, the objective is to nucleate ice crystals in the medium external to cells with the resulting freeze concentration of the solutes causing cells to dehydrate into a glassy state, thus reducing the probability of IIF [1]. This can be achieved after careful selection of cooling rates and aqueous cryoprotective agents (CPAs) [4,5]. However, controlled rate freezing is limited by a dearth of methods to control the onset and location of ice nucleation (IN).

In the absence of controlled IN, the temperature that IN occurs is an unpredictable variable with aqueous CPA solutions typically supercooling well below their equilibrium melting points [6–8]. Ice nucleating at a higher temperature will have longer to form into larger ice crystals, allowing cells to dehydrate and so minimizing IIF. However, ice nucleating at lower temperatures will have less time to form into larger crystals on cooling (at constant cooling rate) [8]. Ice also forms more rapidly when nucleated at lower temperatures and results in a structure containing more, but smaller ice crystals which deviate from the phase diagram. This can cause the ice structure to reform on warming, leading to further cell damage [9,10]. Deep supercooling can reduce viability by increasing the likelihood of lethal IIF during the cooling step [11–13]. When uncontrolled the IN temperature (*T*_nuc_) is determined by the presence (or absence) of heterogeneous ice nucleating sites in the form of impurities within the sample media or features on walls of the vessel [8]. This becomes increasingly problematic when the vessel volume is small (less than 1 ml) as heterogeneous ice nucleating sites are less likely to be present, favouring deep supercooling [8]. The irregular presence of ice nucleating sites within cryopreservation vessels means the degree of supercooling is unpredictable [6,10], often leading to poor and inconsistent post-thaw recovery and function of the preserved cells or tissue. Reliable cryopreservation of plated cell monolayers *in situ* in a high-throughput multiwell plate format would be highly beneficial to the field of drug discovery and toxicity screening. Also, reliably controlled IN would be advantageous in fields such as reproductive medicine and the emerging fields of the banking of complex tissues for transplantation [14,15] and regenerative medicine [16]. However, the typical volume of liquid aliquots in 96-well plates of around 100 µl will supercool by up to 25°C when IN is uncontrolled [8,17]. Currently, isolated cells must be preserved in suspension format before thawing, transferred to multiwell plates and then cultured onto confluent monolayers over several days before meaningful diagnostic assays or manipulations can be performed.

Customary practice for inducing IN during cryopreservation is ‘manual’ nucleation, where touching the outside of the vessel with a very cold object locally cools the contents enough to trigger IN [1,18]. While achievable for vessels such as straws, cryovials and cryobags, it is far less practical for multiwell plates that would require induction of IN in each individual well to be induced simultaneously. A solution to this, where ice-mist falling from a cryogenically cooled object into exposed supercooled liquid in a 96-well plate has been demonstrated [6]. However, having sample contents exposed to air creates sterility issues and scaling up would require some level of standardization and automation and also a requirement for cryogens.

An alternative method of controlling IN is introduction of ice nucleating materials to the cryopreservation medium in a manner that does not interfere with the specimen and which facilitates removal after thawing [8]. Ice nucleating materials are substances that possess sites that raise the temperature at which heterogeneous IN occurs in both supercooled water [19] and aqueous solutions [20]. Ice nucleating materials tested to date include insoluble substances such as silver iodide (AgI) [21,22] and cholesterol [23,24] as well as dispersible substances of biological origin such as Snomax [25–27] and pollen washings [28,29]. Several studies have shown how these ice nucleating materials can be applied to cryopreservation [30–35], although they ultimately may be difficult to make compliant with current good manufacturing practice applications [8] and are less effective at warm temperatures (i.e. contain few nucleating sites) when used in small quantities.

We report here a formulation based on a ‘hyperactive’ variety of potassium (K)-feldspar, ‘LDH1’, as a novel passive ice nucleating material with biocompatibility potential for use with cryopreserving cultured cells *in situ* in 96-well plate format. Mineral powders are emerging as materials that could be used for controlling IN in cryopreservation applications [30,36] and potentially offer advantages over biologically derived ice nucleating materials in terms of their biocompatibility with the samples being preserved. The ice nucleating ability of minerals has been intensely studied [37–39] due to their potential role as ice nucleating particles in atmospheric mineral dust from deserts and their resultant role in modulating the radiative properties of clouds [40,41]. In particular, the minerals K-feldspar, plagioclase feldspar and quartz have since been shown to very effective ice-nucleators [35,37,39] and some ‘hyperactive’ varieties of K-feldspar have been identified which can nucleate ice at temperatures as warm as −2°C [38,42,43]. While the physical reasons for their hyperactivity remain unclear, their active sites are thought to be concentrated on microscopic steps, cracks and pores [43,44]. These sites are unlikely to be proteinaceous ice nucleating contaminants due to their resistance to heat treatments [42,45].

In this study, we characterize the exceptional ice nucleating ability of LDH1 in sub-millilitre aliquots of both pure water and CPA solution in 96-well plates in order to quantitatively compare its ice nucleating activity with other ice nucleating substances applied to this format. We then demonstrate how LDH1 can aid cell cryopreservation *in situ* within multiwell plates using monolayer cultures of immortalized human hepatocytes under small aliquots of CPA. We do this firstly by directly linking *T*_nuc_ of CPA in a 96-well plate to the post-thaw cell recovery rate on an individual well by well basis using remote temperature measurements from infrared (IR) thermography. Secondly, we trial the high throughput cell freezing and recovery performance in several 96-well plates with mammalian cell monolayer cultures frozen with IN controlled by LDH1 delivered in IceStart arrays [8,36].

## 2. Material and methods

### 2.1. Sourcing and preparation of LDH1 and other ice nucleating materials

An ice nucleating mineral-based material—LDH1—was sourced for this study along with four additional materials (two mineral-based and two non-mineral-based) that are already known as having highly active ice nucleating activities and also easily dispersible in water. LDH1 was obtained from a sample of potassium feldspar originating from the Mt Malosa locality, Malawi, a source that has produced samples with similarly exceptional ice nucleating activity [38,44], the mineral phase being previously confirmed as microcline, a polymorph of K-feldspar by X-ray diffraction [36]. Fragments of the LDH1 source material were hand ground into a fine powder using an agate pestle and mortar followed by dry sieving to remove particles above 63 µm in diameter ensuring that particles were evenly suspended in water. The two other mineral samples used were purchased in ground form of similar grain size and these were BCS-376 (a microcline sample without hyperactive ice nucleating sites, Bureau of Analysed Samples, Middlesbrough, UK) and quartz powder (Honeywell Fluka, Cat. No. 83340, purchased from Fischer Scientific, Loughborough, UK). Cholesterol monohydrate crystals were produced by dissolving reagent-grade cholesterol (Sigma Aldrich, Gillingham, UK) in ethanol and recrystallizing as per Sosso *et al.* [24], then grinding the resultant plates into a much finer suspendable powder. Snomax, lyophilized protein pellets that readily disperse into a non-particulate suspension when mixed with water, was obtained from York Snow Inc. (Englewood, CO, USA) and is a commercially available snow-inducer derived from ice nucleating bacteria [25–27].

### 2.2. Droplet freezing assays and derivation of ice nucleating activity

**Figure 1.**
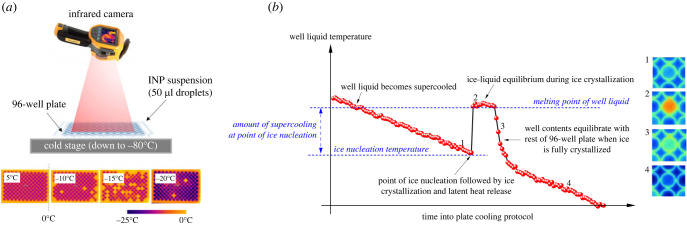
(*a*) Cartoon of IR-NIPI instrument with example IR images of 96-well plate and well ice nucleation events used to obtain all the data in this figure. (*b*) Demonstration of thermal history of well liquid and determination of ice nucleation temperature (*T*_nuc_). Numbers 1–4 correspond to IR images of well at particular points of the thermal history, depicted on the right edge of the figure.

To quantify the ice nucleating activity of LDH1 and other ice nucleators, we performed droplet freezing assays using a combination of two instruments [17,46]. The microlitre Nucleation by Immersed Particle Instrument (µl-NIPI) was used for smaller droplet sizes (1 µl) and Infrared Nucleation by Immersed Particle Instrument (IR-NIPI) for larger droplets on the scale of the working volume of 96-well plates (50 µl). The two instruments differ in the sizes of droplets used and their method of droplet IN temperature detection; however, used in combination they can be used to derive IN site concentrations over several orders of magnitude. The IR-NIPI also has the additional capability to record the thermal history of individual droplets during a cooling run, as outlined in figure 1.

For the µl-NIPI experiments, 30–50 µl sized droplets of IN material suspension were pipetted at room temperature onto a cleaned hydrophobic glass coverslip (HR3–231, Hampton Instruments, Aliso Vejio, USA) resting on an aluminium cooling stage powered by a programmable Stirling engine freezer (Grant-Asymptote EF600, Cambridge, UK). The stage was then enclosed inside a Perspex cell with a dry N_2_ gas flow to prevent frost formation on the coverslip. For each run, the droplets were cooled at a rate of 1°C min^−1^ until all droplets had frozen. Droplet freezing IN events were detected visually with a webcam and the corresponding temperature obtained from the plate temperature thermocouple in the cooling plate.

The larger volume droplet freezing assays were performed on the IR-NIPI instrument where 50 µl droplets of IN material suspended in water (or CPA as required) were pipetted into gamma-ray sterilized 96-well plates (Type 167008, Thermo Scientific Nunclon Delta Surface, Loughborough, UK) and placed onto the cooling plate of a programmable Stirling engine freezer (Asymptote ViaFreeze Research, Cambridge, UK) then subjected to a cooling protocol of 1°C min^−1^ until all droplets were frozen. An IR camera (Fluke Ti9, Everett, USA) mounted above the cooling plate collected images of the 96-well plate every 15 s during freezing runs with each pixel in the images comprising temperature data with the pixel at the centre of each well being defined as the droplet temperature. Harrison *et al*. [17] report the characterized IR temperature measurement. The IR temperature measurement of each well is calibrated using the fact that we know what the temperature of ice–liquid mixture is immediately post freezing (0°C for pure water, lower for solutions; see figure 1). Tests showed agreement between thermocouples embedded in the wells and the calibrated IR temperature measurement. The temperature data contained in each image were then used to construct temperature time series for droplets in each individual well. Droplet IN events in the 96-well plates are detected using the sudden rise in temperature captured by the IR camera resulting from the release of latent heat of crystallization; thus the lowest temperature in the time series just before this ‘jump’ is defined as the droplet IN temperature (figure 1*b*). The latent heat released by a freezing droplet can cause significant warming of droplets in neighbouring wells, so for each run every other well was left empty and filled in ‘chessboard pattern’ so to prevent such artefacts [47]. Following IN the droplet temperature rises then plateaus at what theoretically is the ice–liquid equilibrium at 0°C, providing a point of calibration for each individual well [17]. This method was also used to determine the melting point (or more strictly, the liquidus temperature) of the CPA (−4 ± 0.5°C) from the temperature difference between the ice–liquid equilibrium ‘plateau’ for both water and CPA in wells in the same 96-well plate.

Both the µl-NIPI and IR-NIPI techniques produce data for fraction of droplets frozen as a function of temperature—*f*_ice_(*T*)—for a given aqueous suspension:

2.1 $$f_{\mathrm{ice}}=\frac{n(T)}{N},$$

where *n*(*T*) is the number of droplets frozen at temperature *T* and *N* is the total number of droplets in the assay. Combined with knowledge of the mass of ice nucleator per droplet (*m*), we can derive for any material the density of active sites per unit mass as a function of temperature, *n*_m_(*T*)—a description of the intrinsic ice nucleating activity of a material on a mass-by-mass basis—using the following equation [19,48]:

2.2 $$n_{m}(T)=\frac{-\ln(1-f_{\mathrm{ice}}(T))}{m}.$$

This assumes that sites contained within the material activate at a characteristic temperature without any significant time-dependent component [49]. Using *n*_m_(*T*) allows us to compare the intrinsic ice nucleating activity of particulate samples such as ground mineral powders, normally measured by active sites per INP surface area, *n*_s_(*T*), with non-particulate ice nucleating materials such as Snomax and pollen washing water.

### 2.3. Hepg2 cell culture and 96-well plate cryopreservation with IceStart arrays for ice nucleation control

HepG2 cells, an immortalized human hepatocyte carcinoma cell line, were used in all cryopreservation experiments [50] and procedures were carried out aseptically in a laminar flow hood unless stated. Cells and reagents were obtained from Sigma Aldrich, Gillingham, UK unless stated. Cells were cultured in a humidified incubator at 37°C and 5% CO_2_ in T75 flasks with culture medium (CM) comprised of RPMI 1640 supplemented with 10% fetal bovine serum, 100 kIU l^−1^ penicillin and 0.1 µg l^−1^ streptomycin for 1 week before first passage. Upon each passage up to five (depending on cell yield), 96-well plates pre-coated with rat tail collagen (Roche type 11179179001, 0.05 mg l^–1^ in 0.2% acetic acid) were seeded with cells at a density of 50 000 per well under 200 µl per well of CM and then cultured for 48 h by which time confluent monolayers were achieved. One plate out of each set was assigned as a non-freeze control ‘baseline’ plate while the remaining plates were prepared for cryopreservation. All wells were seeded apart from the edge wells in order to avoid edge effects that are a known source of variability in cell growth when culturing cells in 96-well plates [51,52]. For the experiment to enable the relationship between *T*_nuc_ and cryopreservation survival to be quantified, a single culture plate was cryopreserved using the IR-NIPI instrument. Here every other well in the plate was left empty of cells and CPA, consistent with the droplet freezing assay method described above.

For the high-throughput trials, four plates were cryopreserved—two plates for the immediate post-thaw trial and two plates for the extended 5-day post-thaw trial (see below). Prior to cryopreservation culture medium was replaced with 100 µl per well of a CPA formulation optimized for this cell type, consisting of 10% (v/v) dimethyl sulfoxide and 0.015 M trehalose in CM. The plates were then cooled with ice-bath-cooled aluminium blocks to minimize the toxic effects of the CPA.

For the plate cooled on the IR-NIPI instrument and used in the correlation experiment, a wide range of *T*_nuc_ values were needed. We achieved this by spiking a small number of wells with a few grains of LDH1 powder to nucleate ice close to the melting point and allowing the remaining wells to nucleate in an uncontrolled manner at much lower IN temperatures. For plates used in the high-throughput trials, the individual plates were divided into areas, with each area being subject to a different IN control: IceStart arrays, manual nucleation or uncontrolled nucleation.

**Figure 2.**
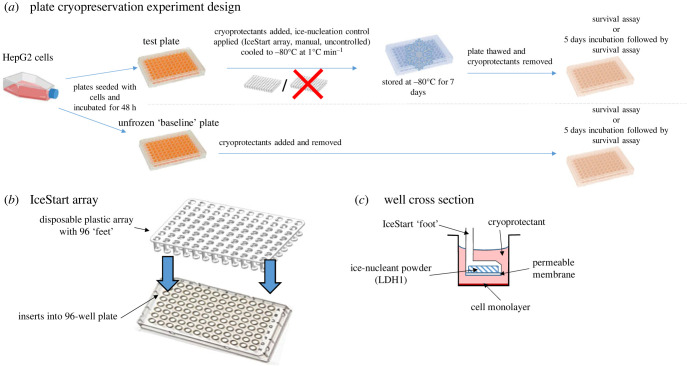
Schematics for (*a*) HepG2 plate cryopreservation protocol and (*b*,*c*) IceStart arrays. Note that IceStart arrays were only used for the high-throughput trials in Results section.

IceStart arrays (figure 2*b*,*c*) are disposable plastic devices, containing a mineral nucleator (in this case LDH1), that can be inserted into a 96-well plate containing adherent cells prior to undergoing freezing and then removed after thawing without contacting the cells. They contain ‘feet’ that slot into each well of the 96-well plate, each of which is loaded with approximately 25 mg of ice nucleating material to trigger IN in the CPA in each well.

The manual nucleation treatment was unsuitable for evaluation in plates that were cultured on after thawing as this method of IN was incompatible with the maintenance of a sterile culture environment. The sections for manual nucleation (if used) and uncontrolled nucleation were covered with sterile plastic strips to prevent potential contamination from IceStart arrays triggering IN. IceStart arrays loaded with LDH1 powder pre-cut into suitably sized portions were wet sterilized by immersion in 70% (v/v) ethanol which was allowed to fully evaporate before the arrays were inserted into their section of the plate. The plates were then sealed and placed on a controlled rate freezer (Asymptote Via Freeze Duo) and cooled at 1°C min^−1^ from +4° to −80°C. The manual nucleation procedure was performed when the plate temperature had dipped below the CPA melting point: the cover strips were removed from the section to be nucleated and then a deep cooled object was held above the plate with the falling ice mist triggering IN in the exposed wells. The technique is described in detail by Daily *et al*. [6]. The plate was then resealed, and the cooling protocol allowed to complete, during which time uncontrolled IN would proceed spontaneously in the remaining plate section. Finally, the plate was transferred to a −80°C freezer and stored for 7 days before thawing.

For thawing, the cryopreserved culture plates were first removed from the −80°C freezer and transferred to a −20°C freezer and held for 20 min to minimize any thermal expansion experienced by the plate upon warming [53]. The plates were then moved into a laminar flow hood, unsealed and placed on aluminium mounting blocks warmed to 37°C, where 100 µl of warm CM was added dropwise to the wells to allow the IceStart arrays' removal once all ice had melted—this procedure typically took no more than 2 min. The CPA was then carefully aspirated away and replaced with 200 µl of CM. Plates used for the immediate post-thaw cell viability trial were then subject to the neutral red viability assay described below, while those used for the 5-day post-thaw culture trial were transferred to a humidified incubator for culture at 37°C and 5% CO_2_ over 5 days with CM replenished after 48 or 72 h.

### 2.4. Cell viability assay and statistical analysis

Viable cell counts were performed using the neutral red dye uptake assay [8,54,55] either immediately after thawing or after 5 days of culture, post-thaw. Cell viability was determined via comparison with that of a non-frozen control plate cultured for 2 days or 7 days, this to provide equivalent time points to the immediate post-thaw and 5-day post-thaw viability assays, respectively. Neutral red was added to culture wells at 50 µg l^−1^ in CM and allowed to equilibrate at 37°C for 2 h, during which time the dye was taken up by viable cells only. The dye solution was then removed from the wells and replaced with a wash-fix solution of 10% (w/v) calcium chloride and 1% (v/v) formaldehyde solution followed by a releasing solution (50% (v/v) ethanol acidified with 1% (v/v) acetic acid). Dye concentration per well was quantified by optical absorbance at 540 nm using a microplate reader (Thermo Scientific Multiskan GO). The absorbance values were translated to cell numbers using a standard curve constructed from neutral red absorbance values for a series of known HepG2 cell concentrations (typically in the range of 10^3^–10^6^ cells per well) from each culture passage.

A pair of high-throughput plated cryopreservation trials were performed which determined cryopreservation success in terms of post-thaw cell viability either immediately post-thaw or after 5 days of post-thaw culture (figure 2*a*). Each of these experiments consisted of eight replicate plates frozen with sections of the plates divided and subject to the varying IN control treatments outlined above. This comprised three sections of 2 × 10 wells for the immediate thaw trial and two sections of 3 × 10 wells for the 5-day trial—we did not include the manual nucleation treatment due to the lack of sterility involved in this procedure. The differences in post-thaw viable cell counts between these groups were analysed by one-way ANOVA with a Games Howell *post hoc* test, required when comparing groups with unequal variances. As the 5-day trial included only two treatments, IceStart and uncontrolled nucleation, comparisons were conducted using a two-sample *t*-test. All statistical significance tests were performed using Minitab v17.20.2. We previously performed preliminary toxicity experiments which showed, using two-sample *t*-tests, neither treatment of the cells with CPA nor IceStart array installation had a significant effect on cell viability. These activities were not regarded as additional treatments. Viable cell numbers were converted to post-thaw recovery rates (or % survival compared to unfrozen controls) by normalizing data to mean viable cell numbers of unfrozen (baseline) plate cultures from the same passage. This was averaged over eight replicate plates for both experimental series.

## 3. Results

### 3.1. The ice nucleating ability of LDH1 compared to other known ice nucleators

**Figure 3.**
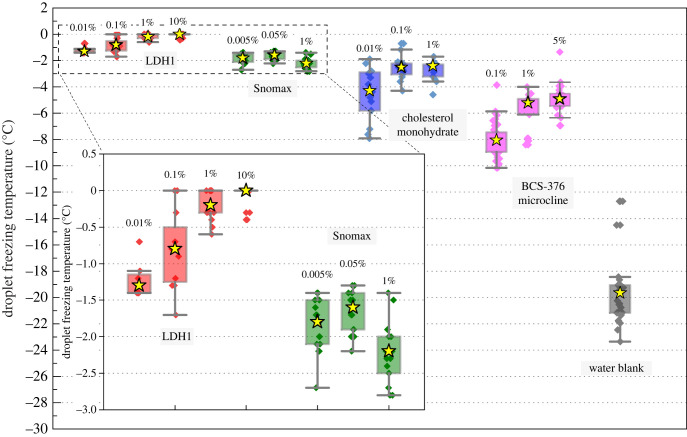
Boxplot showing IR-NIPI results of droplet freezing assays for 50 µl droplets of suspensions of several ice nucleating materials in water at different concentrations. Boxes depict 25th–75th percentiles, bars depict absolute ranges and yellow stars depict median temperatures of droplet freezing.

**Figure 4.**
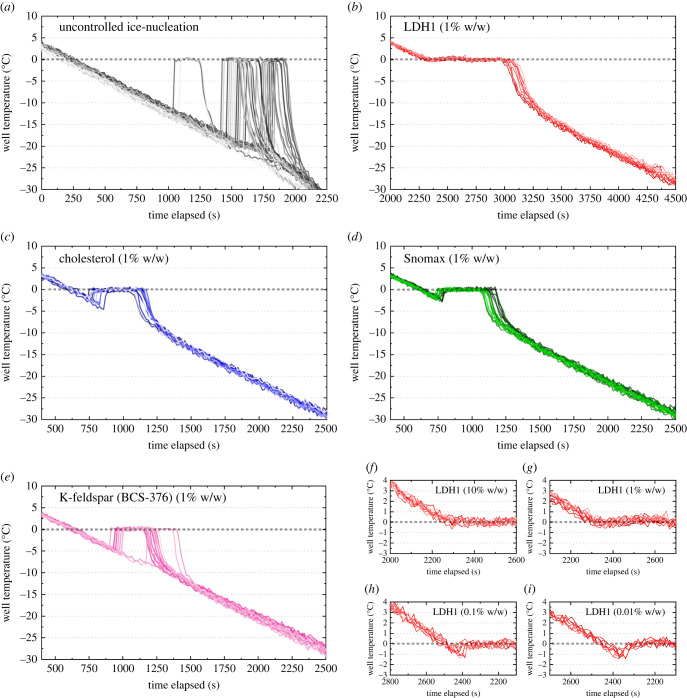
(*a–e*) Thermal history traces of droplet freezing assays shown in figure 3 at concentrations of 1% (w/w). (*f*–*i*) Detailed thermal history of LDH1 runs at all concentrations illustrating the apparent elimination of supercooling. Numbers indicate droplet temperature (°C) on *x*-axis and time into freezing run (s) on *y*-axis.

**Figure 5.**
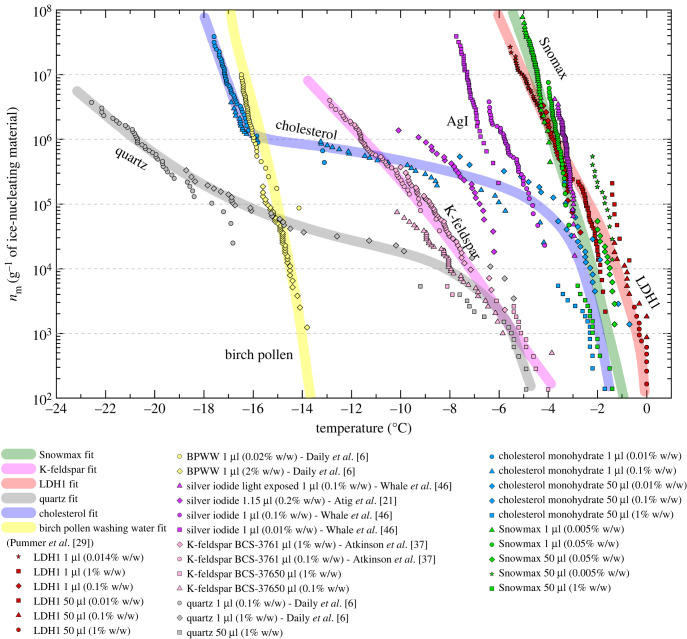
Number of active sites per unit mass as a function of temperature (*n*_m_(*T*)) of LDH1 and other strong ice-nucleators. Data points derived using droplet freezing assays in either 1 µl or 50 µl droplets. Fits for each material were plotted apart from birch pollen which is a parameterization from Pummer *et al.* [29]. No fit was plotted for AgI due to the spread of data points.

To assess the ability of LDH1 to control IN relative to other materials, we performed droplet freezing assays of both 1 µl [42] and 50 µl sized droplets [17] of varyingly concentrated suspensions of this and other potent ice nucleating materials in pure water, cooled at a rate of 1°C min^−1^. First, we focus on the droplet freezing temperatures for 50 µl droplets of ice nucleating materials at various concentrations in 96-well plates, using IR-NIPI (figure 1*a*,*b*), shown as boxplots in figure 3 as a simple comparison of freezing temperatures. For LDH1, the freezing temperatures are warmer than for any other ice nucleating materials and increase with increasing concentration of nucleator (inset in figure 3). Next, the IR-NIPI-derived thermal history of individual wells in 96-well plates containing 50 µl suspension droplets of LDH1 and the other ice nucleating materials, as well as a blank run with pure water, are shown in figure 4*a–i*. In the pure water blank runs, most aliquots of water supercool by more than 15°C before nucleation occurred. The degree of supercooling is clearly much smaller when heterogeneous ice nucleating materials are present. Strikingly however, for wells with LDH1 at suspension concentrations above 1%, none of the well temperatures noticeably dip below 0°C in contrast to the next most active material, Snomax. Instead, they plateau at around 0°C before cooling again after crystallization was complete. This observation implies that no measurable supercooling occurred and IN induced by LDH1 took place close to the melting point,

Using the 1 µl and 50 µl droplet freezing assay data from this study and from the literature, we plot the number of active IN sites per unit mass of material as a function of temperature: *n*_m_(*T*) (figure 5). Figure 5 also shows our *n*_m_(*T*) fits for LDH1 and cholesterol plotted alongside existing literature *n*_m_(*T*) parameterizations for K-feldspar (BCS-376 microcline, non-hyperactive), quartz, birch pollen and Snomax to enable comparison of ice nucleating activity. We found that LDH1 is more ice-active on a mass-by-mass basis than any other mineral, cholesterol or AgI and is comparable to Snomax. Moreover, median freezing temperatures within 1.5°C of the melting point of ice were seen for less than 0.1% LDH1 (0.05 mg of LDH1 per well) suspension droplets in 96-well plates. If LDH1 was able to virtually eliminate supercooling in 50 µl droplets at concentrations as low as 0.1 wt% (0.05 mg of material) it implies that it contains sites able to nucleate ice close to the equilibrium melting point. In principle, the IR-NIPI cannot reliably determine supercooling of less than its nominal uncertainty of ± 1°C [17]. However, the median freezing temperature of the 1 µl droplet assay for LDH1 at 1 wt%, done with the µl-NIPI which has a nominal uncertainty of ±0.4°C [46], was roughly −2°C. This experiment, where droplets contained 50 times less mass of nucleant, therefore serves as a robust lower bound for the true degree of supercooling observed by the IR-NIPI.

The only non-biological materials other than LDH1 previously recorded to induce IN close to the melting point of water are long chain aliphatic alcohol monolayers [56]. This has not previously been seen in any mineral samples either, although compared to other methods such as smaller volume droplet freezing assays [38,57] and differential scanning calorimetry [58] our experiment used a relatively large amount of material in each droplet, meaning we observed the effect of rarer IN sites. Natural mineral samples possess a range of chemical and crystallographic variations that could influence ice nucleating activity [59,60] and as such the reason for the exceptional ice nucleating activity of LHD1 compared to similar K-feldspar samples has yet to be elucidated [42,44]. Snomax, in contrast with LDH1, does not appear to be able to nucleate ice within 1°C of the ice melting point (this is particularly clear in figure 4*d*).

### 3.2. Link between aliquot ice nucleation temperature and post-thaw survival rates of cryopreserved cells

While it is known that ice formation at severe supercooling is likely to be hazardous to cells undergoing cryopreservation, it is not quantitatively clear from the literature whether inducing IN at the melting point has significant benefits [8]. To examine this, we took a 96-well plate containing HepG2 monolayer cultures and, using the IR-NIPI instrument, we simultaneously cryopreserved the cells and recorded the thermal history and IN temperature that occurred in each individual well of the plate. Doing this required a broad range of IN temperatures to occur across the 96-well plate that would be impractical to induce by manual nucleation methods.

**Figure 6.**
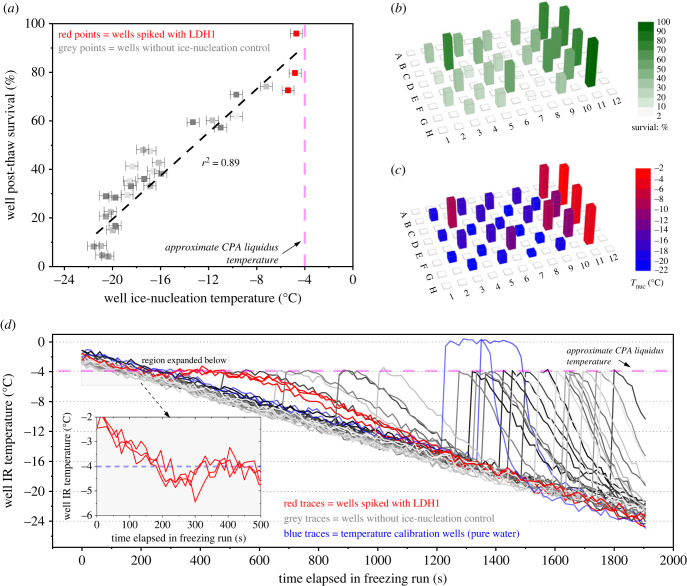
Results for a 96-well plate with HepG2 culture cryopreserved with IR-NIPI instrument showing relationship between *T*_nuc_ and post-thaw cell survival. (*a*) Correlation plot of individual well *T*_nuc_ and post-thaw cell survival values; (*b*) spatial visualization of plate with post-thaw cell survival data; (*c*) spatial visualization of plate with well *T*_nuc_ data; (*d*) thermal history of wells with detail for wells spiked with LDH1. Data for wells with pure water droplets, used for temperature calibration of the IR camera, are also shown.

Using a combination of uncontrolled IN of most wells and spiking a few with LDH1 powder, we were able to produce well *T*_nuc_ values ranging from −21.8°C to −3.4°C in the plate. These clearly correspond to the distribution of plate PTV values (figure 6*a–c*) and the scatter plot of well PTV and *T*_nuc_, shown in figure 6*a*, shows that well IN temperature and PTV are strongly positively correlated (*r*^2^ = 0.89) and appears to show a linear dependence. The thermal histories for each well, shown in figure 6*d*, contrast the temperature fluctuations that occur in wells with IN controlled at warm temperature with those with IN occurring at colder temperature. Figure 6*d* illustrates how the maximum rate of cooling of wells after the ice crystallization phase is far higher for wells with low (uncontrolled) *T*_nuc_ (approx. 7°C min^–1^) than those with *T*_nuc_ near the melting point (3°C min^−1^). The rate of warming upon IN was far faster than the 15 s time interval we used for temperature measurements apart from when nucleation occurred close (within 1°C) to the melting point. For wells nucleating below −10°C the rise to melting point appeared instantaneous implying warming rates of hundreds of °C per minute. Overall, it is clear that warmer well IN temperatures and corresponding smaller rates of change of temperature are conducive to cell survival.

### 3.3. High-throughput cryopreservation of HepG2 cell monolayers *in situ* within 96-well plate with LDH1 delivered via IceStart arrays

We then tested our method of 96-well plate cell cryopreservation with controlled IN using IceStart arrays as vehicle for delivery of LDH1 [36,61]. This strategy enabled us to determine whether LDH1 significantly improved cryopreservation performance using a protocol that could be scaled up. We were unable to measure IN temperatures for the 96-well plates cryopreserved in this section; however, each foot of the IceStart array contains 25 mg of LDH1, which is considerably greater than the amounts that reliably eliminated supercoolings of more than 1°C in the IR thermography experiments. We can therefore assume that IN was initiated close to the CPA melting point wherever IceStart arrays were applied to cryopreserved 96-well plates.

We performed two 96-well plate freezing trials, each of which comprised eight replicate plates of HepG2 monolayer cultures cryopreserved with different methods of IN control. Post-thaw cell viability (PTV, number of viable cells) was assessed immediately upon thawing and was compared to that of a non-frozen control 96-well plate culture to determine acute cell post-thaw survival rates (PTS, %). The first trial determined PTV immediately upon thawing, while for the second trial the cryopreserved plates were cultured for a further 5 days after thawing to determine longer term cell viability and performance. Plates were frozen with portions divided into varying methods of IN control: IceStart arrays loaded with LDH1 powder, uncontrolled nucleation as a negative control and manual nucleation as a positive control. The manual nucleation method used ice mist falling from a cold object and has been previously demonstrated to induce IN uniformly at moderately low supercooling (at most 5°C) during similar experiments with 96-well plates [6]. However, as this method involves exposing the plate to ambient air under non-sterile conditions, this method was unsuitable for inclusion in the 5-day post-thaw culture trial.

**Figure 7.**
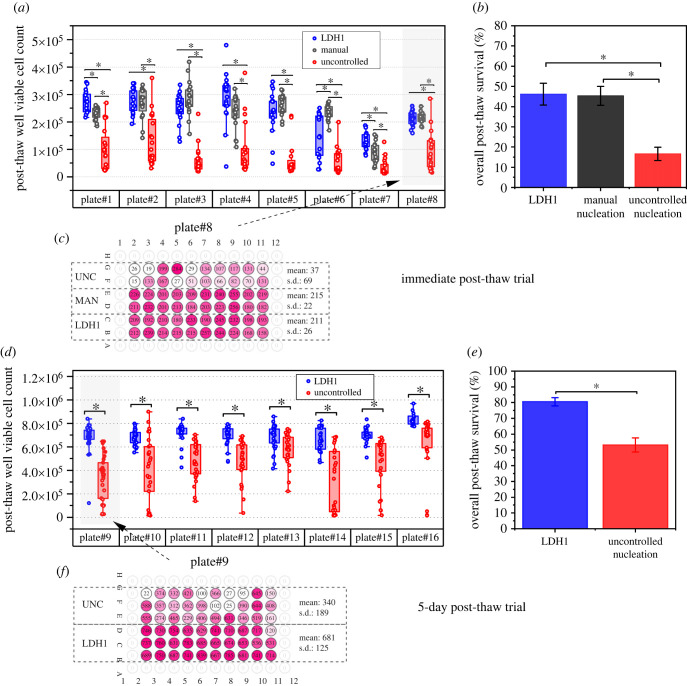
Results for high-throughput trials of monolayers of HepG2 cells cryopreserved in 96-well plate with and without controlled IN. Viability assays performed immediately post-thaw (*a–c*) and 5 days post-thaw (*d*–*f*). Example results for individual plates showing layout of IN method and results are shown in (*c*) and (*f*) with numbers denoting thousands of cells and colour strength indicating post-thaw viability (s.d. = standard deviation). Boxplots and ANOVA results for individual freezing runs are shown in (*a*) and (*d*). Overall post-thaw survival rates averaged over all eight runs are shown in (*b*) and (*e*). Note that manual nucleation was not used in the 5-day trial as this technique could not be executed in sterile conditions. Error bars indicate standard error of mean; bars and asterisk (*) indicate significance between groups was *p* < 0.05.

The results of the freezing trials for both immediate post-thaw and 5-day post-thaw, along with statistical significance testing (one-way ANOVA for the immediate post-thaw trial and two-sample *t*-test for the 5-day post-thaw trial) are shown in figure 7. For the immediate post-thaw viability assessment, the wells of all eight plates’ wells with IceStart applied had significantly higher PTV cell counts than where IN was uncontrolled (*p* < 0.05). Differences were seen in individual plates between IceStart and manual nucleation wells; however, overall neither nucleation method resulted in constantly higher or lower results. The overall immediate post-thaw recovery rates of the eight replicates were 46.2%, 45.3% and 16.6% using IceStart, manual nucleation and uncontrolled nucleation respectively, meaning both IceStart and manual nucleation resulted in significantly higher PTS than uncontrolled nucleation. There was no significant difference in outcome between the IceStart and manual nucleation methods (one-way ANOVA, *p* < 0.05). For the 5-day post-thaw trial, where only IceStart and uncontrolled nucleation methods of IN were used, all 8 replicate plates showed significantly higher PTV cell number with IceStart deployed. Overall PTS over all plates averaged: 80.5% versus 53.1%, respectively (two-sample *t*-test, *p* < 0.05). In contrast to the immediate post-thaw viability trial, when the cryopreserved plates were cultured for a further 5 days after thawing the data were far more variable. For example, the uncontrolled nucleation datasets included clusters of ‘dead’ wells and ‘recovered’ wells within plates: this is clearest in plate#10, #14 and #15 (figure 7*e*). In contrast, the LDH1 nucleated wells have a much greater and less variable PTV.

**Figure 8.**
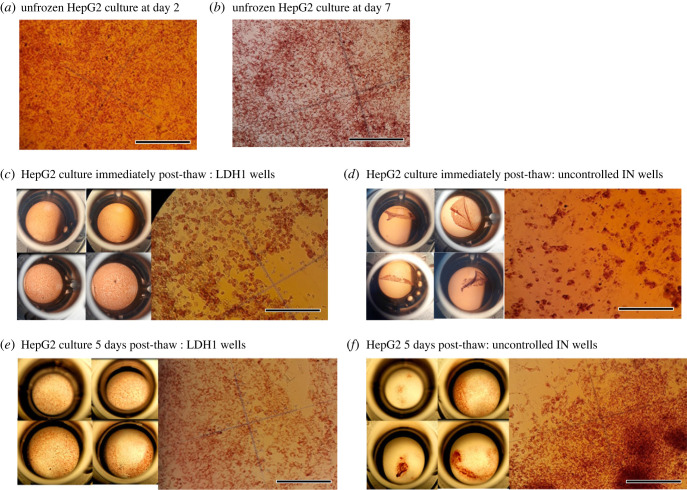
Microscope images of HepG2 cell monolayers after neutral red staining illustrating differences in cell morphology dependent on IN control. Low magnification images of whole wells on left of (*c*–*f*) illustrate condition of monolayer (well diameter is 6.4 mm for indication of scale). Higher magnification images on right of (*c*–*f*) illustrate condition of individual cells. Scale bar represents 200 µm.

Using an inverted light microscope, we visually inspected the cell monolayers in the 96-well plates of non-frozen control cultures compared with those after thawing at the point where neutral red dye had stained all the viable cells. Lower magnification images detailing the condition of the monolayers and higher magnification images resolving individual cells are shown in figure 8*c–f*. Analysis revealed that the HepG2 monolayer detached from the culture plate surface when IN was not controlled, although the degree of detachment was variable and rarely complete. This detachment was almost absent when IceStart or manual nucleation was used (figure 8*c*). In the cases where the cell layers had detached, there were cells still visibly stained by the dye and therefore still viable. Closer inspection of the monolayer (where still *in situ*) at the immediate post-thaw stage revealed a network of spherical viable cells that had lost their epithelial morphology and were clumped together in places together with dead (unstained) cells (figure 8*d*). The ratio of viable cells to dead cells was qualitatively lower where IN was uncontrolled compared to controlled, either by IceStart or manual nucleation. Re-examination after 5 days of culture post-thaw (figure 8*e*,*f*) showed the surviving cells had re-attached and started proliferating once again, consistent with the general higher cell viability seen for all treatment groups compared with immediate post-thaw. The manner of proliferation in wells frozen with controlled IN versus uncontrolled IN was, however, distinct and reflected the variable amount of initial post-thaw monolayer detachment where well cultures grew as confluent monolayers compared with irregular masses.

## 4. Discussion

### 4.1. How does eliminating supercooling protect cryopreserved cells?

Our observations quantitatively support the generally accepted principle that for slow-freezing cryopreservation of cells, avoidance of severe supercooling is required for good cell post-thaw recovery and functionality [12,32,62,63]. The nucleation and growth of extracellular ice enable migration of water from the cell, raising the concentration of solutes in the cell and therefore raising the glass transition temperature of the unfrozen cell contents. This allows the cell interior to solidify without IIF occurring [64], because in the absence of extracellular ice, IIF is more likely to occur due to higher water content of the cells [1]. The configuration of cells in this case—monolayers attached to a substrate—is prone to ice propagation through cell-to-cell contact making them especially sensitive to incidence of IIF [11]. Another possible protective mechanism from warm IN temperature is the prevention of IIF occurring during thawing. The degree of supercooling affects the ice crystal properties that result after freezing with deeper supercooling leading to a finer-grained network [8]. Smaller crystals have higher interfacial free energy which can drive ice recrystallization ultimately leading to IIF. Warmer IN resulting in a coarser-grained extracellular ice crystal network has previously been demonstrated to prevent this [65].

Detachment of the HepG2 cell monolayer from the surface of culture plate appeared to result in low post-thaw viability and mostly only occurred when IN was left uncontrolled (figure 8). Monolayer detachment is a known issue from previous attempts to cryopreserve cells in the multiwell plate format. When using a similar protocol to cryopreserve primary cultures of bovine granulosa in a 96-well plate using manual versus uncontrolled IN a comparable situation was seen with cell layer detachment being more apparent in the latter [6]. By contrast, the prevention of monolayer detachment was likely related to controlling IN in the well liquid at close to the melting point as this prevents the severe temperature fluctuations that would otherwise occur inside the well following the ice crystallization process. In the case where a plate culture was cryopreserved coincident with IR temperature measurements (figure 6) we calculated that the maximum cooling rates experienced in the warmest nucleating wells during thermal equilibration after latent heat release were around 3°C min^−1^ compared with up to 7°C min^−1^ in the most deeply supercooled wells. This resulted in an effective cooling rate much higher than the nominal 1°C min^−1^ defined by the cooling programme on the controlled rate freezer. Not only do higher than optimum cooling rates after extracellular ice formation mean that cells cannot dehydrate fast enough to avoid IIF [1], but also that the frozen cell monolayer and substrate thermally contract at different rates causing the cells to lose their adhesion to the surface matrix of the plate [66,67]. Eskandari *et al*. [66] and Rutt *et al*. [67] were able to prevent monolayers of various cell types detaching after cryopreservation by using a polyvinyl chloride polymer, which has a thermal expansion coefficient close to that of ice, as a substrate. Alternatively, the cell layer may have been mechanically overstressed and detached by differential expansion caused by the latent heat release at the point of IN rather than the cooling and contraction during the equilibration step. Ice nucleation at deeper levels of supercooling results in correspondingly higher effective rates of heating and cooling [6] but, as we observed here, the increase in heating rates with *T*_nuc_ is far more extreme than that of the cooling rates. In summary, severe temperature fluctuations following IN events are a plausible reason for the cell monolayer detachments and were minimized by controlling IN at the melting point with IceStart arrays. This may negate the need for using thermally matched substrate materials for cell monolayer freezing.

## 5. Concluding remarks

Overall, we have demonstrated how the survival of cell monolayers frozen within multiwell plates is directly related to the temperature of IN and how it can be significantly improved with the aid of a mineral-based ice nucleating agent. A sterilizable device inserted into the 96-well plate passively and reliably induces IN at close to the melting point of the host cryoprotectant, thus avoiding severe thermal stresses caused by rapid latent heat release associated with ice crystal growth from deeply supercooled liquid. *T*_nuc_ is difficult to control in a repeatable way [8], and it has been stated that a downside to using chemical ice nucleating agents is not being able to control the temperature of IN reliably and precisely since nucleation is inherently a probabilistic process [68]. Hence, our use of LDH1, where supercooling is consistently almost eliminated and there is therefore no variation in IN temperatures, represents a significant advance. Also, the exceptional ice nucleating activity of LDH1 that we report here allows us to use only very small amounts (1 mg, or even less) of it to virtually eliminate aqueous supercooling or, according to the limits of the methodology used here, certainly limiting it to at most 2°C.

Our method for quantifying the relationship between *T*_nuc_ and cell viability described in this work could be adapted to other cell or tissue types and biological markers as well as other multiwell plate formats (e.g. 384-well plates). This will enable examination of the importance of IN control for cryopreservation of other cell types and biological entities. This might enable existing cryopreservation protocols to be suitably tailored. Further research is needed to identify the nature of the hyperactive ice nucleating sites found in mineral specimens such as LDH1 to enable further sources of this material to be identified or manufactured. More detailed studies are required to determine improved delivery methods for LDH1 (or similar mineral nucleating agents), such as encapsulation or integration into vessel surfaces [14]. Moreover, the exceptional ice nucleating activity of hyperactive ice nucleating minerals such as LDH1 means that IN can potentially be controlled at even smaller scales such as 384-well plates or in microfluidic droplets [69,70]. This possibility creates new scope for even higher throughput sample cryopreservation in new applications in which IN can be controlled.

## Data Availability

The data underlying the study have been uploaded to a publically accessible online data repository hosted by the University of Leeds (https://doi.org/10.5518/1288).
